# Draft Genome Sequence of Enterococcus faecium CL-6729, a Clinical Isolate Showing Constitutive Vancomycin Resistance

**DOI:** 10.1128/MRA.00888-18

**Published:** 2018-11-21

**Authors:** Beatriz Nascimento Monteiro da Silva, Adriana Rocha Faria, Stephanie da Silva Rodrigues de Souza, Paul J. Planet, Vânia Lúcia Carreira Merquior, Lúcia Martins Teixeira

**Affiliations:** a Faculdade de Ciências Médicas, Universidade do Estado do Rio de Janeiro, Rio de Janeiro, Brazil; b Instituto de Microbiologia Paulo de Góes, Universidade Federal do Rio de Janeiro, Rio de Janeiro, Brazil; c Perelman School of Medicine, University of Pennsylvania, Philadelphia, Pennsylvania, USA; d Pediatric Infectious Disease Division, Children’s Hospital of Philadelphia, Philadelphia, Pennsylvania, USA; e Sackler Institute for Comparative Genomics, American Museum of Natural History, New York, New York, USA; University of Arizona

## Abstract

Here, we present the draft genome sequence of an unusual Enterococcus faecium isolate (CL-6729) showing constitutive expression of the VanA type of vancomycin resistance. The isolate was recovered from a patient with a nosocomial urinary tract infection in Brazil.

## ANNOUNCEMENT

Enterococcus faecium has emerged as an important multidrug-resistant nosocomial pathogen. Hospital-associated clones of this microorganism are characterized by the acquisition of mobile genetic elements, including determinants of acquired vancomycin resistance (1–3). Strains with the VanA phenotype possess inducible high-level resistance to both vancomycin and teicoplanin, mediated by the *vanA* gene cluster (*vanSRHAXYZ*) found on transposon Tn*1546* (4). Here, we report the genome sequence of an unusual E. faecium clinical isolate (CL-6729) showing constitutive expression of the VanA phenotype. The isolate was recovered from a patient with a nosocomial urinary tract infection in Rio de Janeiro, Brazil.

A single bacterial colony grown on a blood agar plate was inoculated into 5 ml of tryptic soy broth and incubated overnight at 37°C. Genomic DNA was extracted from 1.5 ml of that culture by using a Wizard genomic DNA purification kit (Promega, Madison, WI, USA), prepped using the Nextera XT kit, and sequenced on a HiSeq 2500 sequencer (Illumina, Inc., San Diego, CA, USA) with 125-bp paired-end reads. Sequencing resulted in 1,429,676 paired-end Illumina reads and 178,709,500 bp that were trimmed by using Trimmomatic 0.36 (5) with the following parameters: sliding window, 4:20; leading, 25; trailing, 25; headcrop, 18; crop, 110; and minlength, 36. High-quality reads were assembled and annotated using resources of the Pathosystems Resource Integration Center (PATRIC 3.4.11) with default parameters (6). Antimicrobial resistance and virulence genes, insertion sequences (IS), plasmids, and prophages were identified by using the following Web-available tools with default parameters: ResFinder (7), VirulenceFinder (8), ISfinder (9), PlasmidFinder (10), and PHAST (11).

The draft genome of E. faecium CL-6729 consists of 194 contigs (*N*_50_, 35,510 bp) with a coverage of 39×. The genome size was 2,902,384 bp with a G+C content of 37.70%. Three rRNAs, 44 tRNAs, and 3,002 coding sequences (CDS) were identified. Most of the *vanA* locus genes located on Tn*1546* were intact. An alignment with sequences for Tn*1546* (GenBank accession number M97297) and for IS commonly found in E. faecium showed the presence of IS*19* (GenBank accession number AF169285) located upstream of the first 119 bp of the *vanS* gene, suggesting that the constitutive expression of vancomycin resistance could be due to the impairment of the sensor protein VanS. This concept was also supported by real-time PCR analysis of gene expression (our unpublished data).

The following antimicrobial resistance genes were also identified: *ant(6)-Ia*, *aac(6′)-aph(2″)*, *aph(3′)-III*, and *sat-4*, associated with aminoglycoside resistance; and *msr*(C) and *erm*(B), related to macrolide resistance. Alignment of the *parC* and *gyrA* genes with the E. faecium DO genome (GenBank accession number CP003583) and QRDR regions (GenBank accession numbers AF060881 and AB017811) revealed single amino acid polymorphisms in codons 82 (Ser to Ile) and 84 (Ser to Arg). A comparison of the predicted C- and N-terminal regions of PBP5 (coded by the *pbp5* gene) with a reference sequence, GenBank accession number X84860, showed the Met-485-Ala substitution, which is associated with high-level resistance to β-lactam antibiotics (12). Three virulence-related determinants were detected, *acm*, *efaAfm*, and *esp*, which are associated with E. faecium emergence as a nosocomial pathogen (1, 13, 14).

Besides IS*19*, another 17 insertion sequences were identified, including the IS*16* marker, which has been associated with hospital isolates (15, 16). Moreover, PHAST predicted one intact prophage (PHAGE_Lister_2389, GenBank accession number NC_003291) and two incomplete prophage regions (PHAGE_Entero_vB_IME197, GenBank accession number NC_028671; PHAGE_Paenib_Xenia, GenBank accession number NC_028837). Plasmids of the repUS15 and rep17 families were also detected. Multilocus sequence typing (MLST) characterized this strain as sequence type 78 (ST78) (http://pubmlst.org/efaecium), lineage 78 (BAPS 2-1) (13).

### Data availability.

This whole-genome shotgun project has been deposited at DDBJ/ENA/GenBank under the accession number QMDJ00000000. The version described in this paper is the first version, QMDJ01000000. Raw sequence reads have been deposited in the NCBI Sequence Read Archive (SRA) under the BioProject accession number PRJNA477638.
